# Supplementary material for: Total energy expenditure is repeatable in adults but not associated with short-term changes in body composition
Source: Nat Commun. 2022 Jan 10;13:99. doi: 10.1038/s41467-021-27246-z (PMC8748652; doi:10.1038/s41467-021-27246-z)
Supplement: Supplementary file 1 — Supplementary Information [file 41467_2021_27246_MOESM1_ESM.pdf]

Supplementary Information

Total energy expenditure is repeatable in adults but not associated with changes in body composition

Rebecca Rimbach[1,2]*†, Yosuke Yamada[3,4]*, Hiroyuki Sagayama[5]*, Philip N. Ainslie[6], Lene F. Anderson[7], Liam J. Anderson[6,8], Lenore Arab[9], Issaad Baddou[10], Kweku Bedu-Addo[11], Ellen E. Blaak[12], Stephane Blanc[13,14], Alberto G. Bonomi[15], Carlijn V.C. Bouten[16], Pascal Bovet[17], Maciej S. Buchowski[18], Nancy F. Butte[19], Stefan G.J.A. Camps[12], Graeme L. Close[6], Jamie A. Cooper[13], Sai Krupa Das[20], Lara R. Dugas[21], Ulf Ekelund[22], Sonja Entringer[23,24], Terrence Forrester[25], Barry W. Fudge[26], Annelies H. Goris[12], Michael Gurven[27], Catherine Hambly[28], Asmaa El Hamdouchi[10], Marije B. Hoos[12], Sumei Hu[29] , Noorjehan Joonas[30], Annemiek M. Joosen[12], Peter Katzmarzyk[31], Kitty P. Kempen[12], Misaka Kimura[3], William E. Kraus[32], Robert F. Kushner[33], Estelle V. Lambert[34], William R. Leonard[35], Nader Lessan[36], Corby K. Martin[31], Anine C. Medin[7,37], Erwin P. Meijer[12], James C. Morehen[38,6], James P. Morton[6], Marian L. Neuhouser[39], Theresa A. Nicklas[19], Robert M. Ojiambo[40,41], Kirsi H. Pietiläinen[42], Yannis P. Pitsiladis[43], Jacob Plange-Rhule[44**], Guy Plasqui[45], Ross L. Prentice[39], Roberto A. Rabinovich[46], Susan B. Racette[47], David A. Raichlen[48], Eric Ravussin[31], Rebecca M. Reynolds[49], Susan B. Roberts[20], Albertine J. Schuit[50], Anders M. Sjödin[51], Eric Stice[52], Samuel S. Urlacher[53], Giulio Valenti[12], Ludo M. Van Etten[12], Edgar A. Van Mil[54], Jonathan C. K. Wells[55], George Wilson[6], Brian M. Wood[56,57], Jack Yanovski[58], Tsukasa Yoshida[5], Xueying Zhang[28,29], Alexia J. Murphy-Alford[59], Cornelia U. Loechl[59], Amy H. Luke[60]*, Jennifer Rood[31]*, Dale A. Schoeller[61]*, Klaas R. Westerterp[62]*, William W. Wong[19]*, John R. Speakman[63,28,29,64]*†, Herman Pontzer[1,65]*† and the IAEA DLW Database Consortium§

*Corresponding author. Email: rrimbach@gmail.com (R.R.); herman.pontzer@duke.edu (H.P.); j.speakman@abdn.ac.uk (J.R.S.); yyamada831@gmail.com (Y.Y.); sagayama.hiroyuki.ka@u.tsukuba.ac.jp (H.S.); aluke@luc.edu (A.H.L.); jennifer.rood@pbrc.edu (J.R.); dschoell@nutrisci.wisc.edu (D.A.S.); k.westerterp@maastrichtuniversity.nl (K.R.W.); wwong@bcm.edu (W.W.W.)
† These authors contributed equally
** deceased
§IAEA DLW Database Consortium members are listed in the supplementary materials.

1. Evolutionary Anthropology, Duke University, Durham NC, USA
2. School of Animal, Plant & Environmental Sciences, University of the Witwatersrand, Johannesburg, South Africa
3 National Institute of Health and Nutrition, National Institutes of Biomedical Innovation, Health and Nutrition, Tokyo, Japan.
4 Institute for Active Health, Kyoto University of Advanced Science, Kyoto, Japan.
5 Faculty of Health and Sport Sciences, University of Tsukuba, Ibaraki, Japan.
6 Research Institute for Sport and Exercise Sciences, Liverpool John Moores University, Liverpool, UK.

7 Department of Nutrition, Institute of Basic Medical Sciences, University of Oslo, 0317 Oslo, Norway.
8 Crewe Alexandra Football Club, Crewe, UK.
9 David Geffen School of Medicine, University of California, Los Angeles.
10 Unité Mixte de Recherche en Nutrition et Alimentation, CNESTEN- Université Ibn Tofail URAC39, Regional Designated Center of Nutrition Associated with AFRA/IAEA.
11 Department of Physiology, Kwame Nkrumah University of Science and Technology, Kumasi, Ghana.
12 Maastricht University, Maastricht, The Netherlands.
13 Nutritional Sciences, University of Wisconsin, Madison, WI, USA
14 Institut Pluridisciplinaire Hubert Curien. CNRS Université de Strasbourg, UMR7178, France.
15 Phillips Research, Eindhoven, The Netherlands.
16 Department of Biomedical Engineering and Institute for Complex Molecular Systems Eindhoven Unversity of Technology, Eindhoven, The Netherlands
17 Institute of Social and Preventive Medicine, Lausanne University Hospital, Lausanne, Switzerland.
18 Division of Gastroenterology, Hepatology and Nutritiion, Department of Medicine, Vanderbilt University, Nashville, Tennessee, USA
19 Department of Pediatrics, Baylor College of Medicine, USDA/ARS Children's Nutrition Research Center, Houston, Texas, USA.
20 Jean Mayer USDA Human Nutrition Research Center on Aging, Tufts University. 711 Washington St. Boston MA 02111
21 Department of Public Health Sciences, Parkinson School of Health Sciences and Public Health, Loyola University, Maywood, IL, USA.
22 Department of Sport Medicine, Norwegian School of Sport Sciences, Oslo, Norway.
23 Charité – Universitätsmedizin Berlin, corporate member of Freie Universität Berlin, Humboldt-Universität zu Berlin, and Berlin Institute of Health (BIH), Institute of Medical Psychology, Berlin, Germany.
24 University of California Irvine, Irvine, California, USA.
25 Solutions for Developing Countries, University of the West Indies, Mona, Kingston, Jamaica.
26 University of Glasgow, Glasgow, UK.
27 Department of Anthropology, University of California Santa Barbara, Santa Barbara, CA, USA.
28 Institute of Biological and Environmental Sciences, University of Aberdeen, Aberdeen, UK
29 State Key Laboratory of Molecular Developmental Biology, Institute of Genetics and Developmental Biology, Chinese Academy of Sciences, Beijing, China
30 Central Health Laboratory, Ministry of Health and Wellness, Mauritius.
31 Pennington Biomedical Research Center, Baton Rouge, Louisiana, USA.
32 Department of Medicine, Duke University, Durham, North Carolina, USA.
33 Northwestern University, Chicago, IL, USA.
34 Research Unit for Exercise Science and Sports Medicine, University of Cape Town, Cape Town, South Africa.
35 Department of Anthropology, Northwestern University, Evanston, IL, USA.
36 Imperial College London Diabetes Centre, Imperial College London.
37 Department of Nutrition and Public Health, Faculty of Health and Sport Sciences, University of Agder, 4630 Kristiansand, Norway.
38 The FA Group, Burton-Upon-Trent, Staffordshire, UK.
39 Division of Public Health Sciences, Fred Hutchinson Cancer Research Center and School of Public Health, University of Washington, Seattle, WA, USA.
40 Moi University, Eldoret, Kenya.
41 University of Global Health Equity, Rwanda.
42 Helsinki University Central Hospital, Helsinki, Finland.
43 University of Brighton, Eastbourne, UK.
44 Department of Physiology, Kwame Nkrumah University of Science and Technology, Kumasi, Ghana

45 Department of Nutrition and Movement Sciences, Maastricht University, Maastricht, The Netherlands.
46 University of Edinburgh, Edinburgh, UK.
47 Program in Physical Therapy and Department of Medicine, Washington University School of Medicine, St. Louis, Missouri, USA.
48 Biological Sciences and Anthropology, University of Southern California, California, USA.
49 Centre for Cardiovascular Sciences, Queen's Medical Research Institute, University of Edinburgh, Edinburgh, UK.
50 University of Tilburg, Tilburg, The Netherlands
51 Department of Nutrition, Exercise and Sports, Copenhagen University, Copenhagen, Denmark.
52 Stanford University, Stanford CA, USA.
53 Department of Anthropology, Baylor University, Waco, TX, USA.
54 Maastricht University, Maastricht and Lifestyle Medicine Center for Children, Jeroen Bosch Hospital's-Hertogenbosch, The Netherlands.
55 Population, Policy and Practice Research and Teaching Department, UCL Great Ormond Street Institute of Child Health, London, UK.
56 University of California Los Angeles, Los Angeles, USA.
57 Max Planck Institute for Evolutionary Anthropology, Department of Human Behavior, Ecology, and Culture, Germany.
58 Growth and Obesity, Division of Intramural Research, NIH, Bethesda, MD, USA.
59 Nutritional and Health Related Environmental Studies Section, Division of Human Health, International Atomic Energy Agency, Vienna, Austria.
60 Division of Epidemiology, Department of Public Health Sciences, Loyola University School of Medicine, Maywood Illinois, USA.
61 Biotech Center and Nutritional Sciences University of Wisconsin, Madison, Wisconsin, USA.
62 School of Nutrition and Translational Research in Metabolism, University of Maastricht, Maastricht, The Netherlands.
63 Center for Energy Metabolism and Reproduction, Shenzhen Institutes of Advanced Technology, Chinese Academy of Sciences, Shenzhen, China
64 CAS Center of Excellence in Animal Evolution and Genetics, Kunming, China.
65 Duke Global Health Institute, Duke University, Durham, NC, USA

**Supplementary Note 1**	Page 5
**Supplementary Fig. 1.**	Page 6
**Supplementary Fig. 2.**	Page 7
**Supplementary Note 2**	Page 8
**Supplementary Fig. 3.**	Page 9
**Supplementary Table 1.**	Page 11
**The IAEA DLW Database Consortium**	Page 12

Supplementary Note 1

Repeatability using adjusted total energy expenditure (TEE)

We computed a predicted total energy expenditure TEE from a multiple regression model with TEE as the dependent variable and FFM, FM, age, and sex as independent variables. We calculated adjusted TEE by dividing observed TEE by predicted TEE and multiplying the value by 100. Thus, an adjusted TEE of 120% indicates an observed measured TEE that is 20% greater than predicted from body composition variables. Adults had an average ± SD adjusted TEE1/TEE2 ratio of 1.006 ± 0.042 (range: 0.814 – 1.203). Adults aged 20-60 y had a ratio of 1.002 ± 0.034 (range: 0.902 – 1.207) and children had a ratio of 1.004 ± 0.085 (range: 0.754 – 1.261). Repeatability of TEE (estimated as adjusted TEE1/TEE2) did not change with increasing time between both TEE measurements (Supplementary Fig. 1a-c).

Confirming the results of the mixed effects modeling approach, adjusted TEE1 correlated with adjusted TEE2 (Pearson's product-moment correlation: $r_p = 0.63$, $P < 0.0001$) for all adults (Supplementary Fig. 1d) and for the subset of adults aged 20 – 60 y (Pearson's product-moment correlation: $r_p = 0.69$, $P < 0.0001$; Supplementary Fig. 1e). In contrast, adjusted TEE1 did not correlate with adjusted TEE2 (Pearson's product-moment correlation: $r_p = 0.07$, $P = 0.55$) in children (Supplementary Fig. 1f). The difference between adjusted TEE1 and adjusted TEE2 was statistically significant for the entire adult sample (Paired t test: t = 2.472, df = 347, P = 0.013, Cohen’s d = 0.13 (95% CI = 0.01 – 0.23); Supplementary Fig. 1g), but not for adults aged 20 – 60 y (t = 0.867, df = 266, P = 0.38, Cohens’ d = 0.05 (95% CI = -0.06 – 0.18); Supplementary Fig. 1h) or for children (Paired t test for children with 2 measurements: t = -0.906, df = 26, P = 0.373, Cohens d = -0.17 (95% CI = -0.5 – 0.25); repeated measures ANOVA for children with 3 measurements: F = 0.59, P = 0.45; Supplementary Fig. 1i). While some comparisons of TEE1 and TEE2 achieved statistical significance, the differences were notable for being small. The difference in adjusted TEE between repeated measurements for each subject was 0.46 ± 4.24% (range: -22.7 – 16.9%), and the absolute difference was 3.08 ± 2.94% (range: 0.001 – 22.7%) in adults. The difference in adjusted TEE between repeated measurements at ages 2 and 4 was -0.87 ± 10.5% (range: -29.9 – 15.4%) for each subject and the absolute difference was 8.01 ± 6.76% (range: 0.02 – 29.9%). The difference in adjusted TEE was -0.02 ± 9.4% (range: -32.7 – 20.7%) between ages 4 and 6 and the absolute difference was 6.46 ± 6.76% (range: 0.15 – 32.7%).

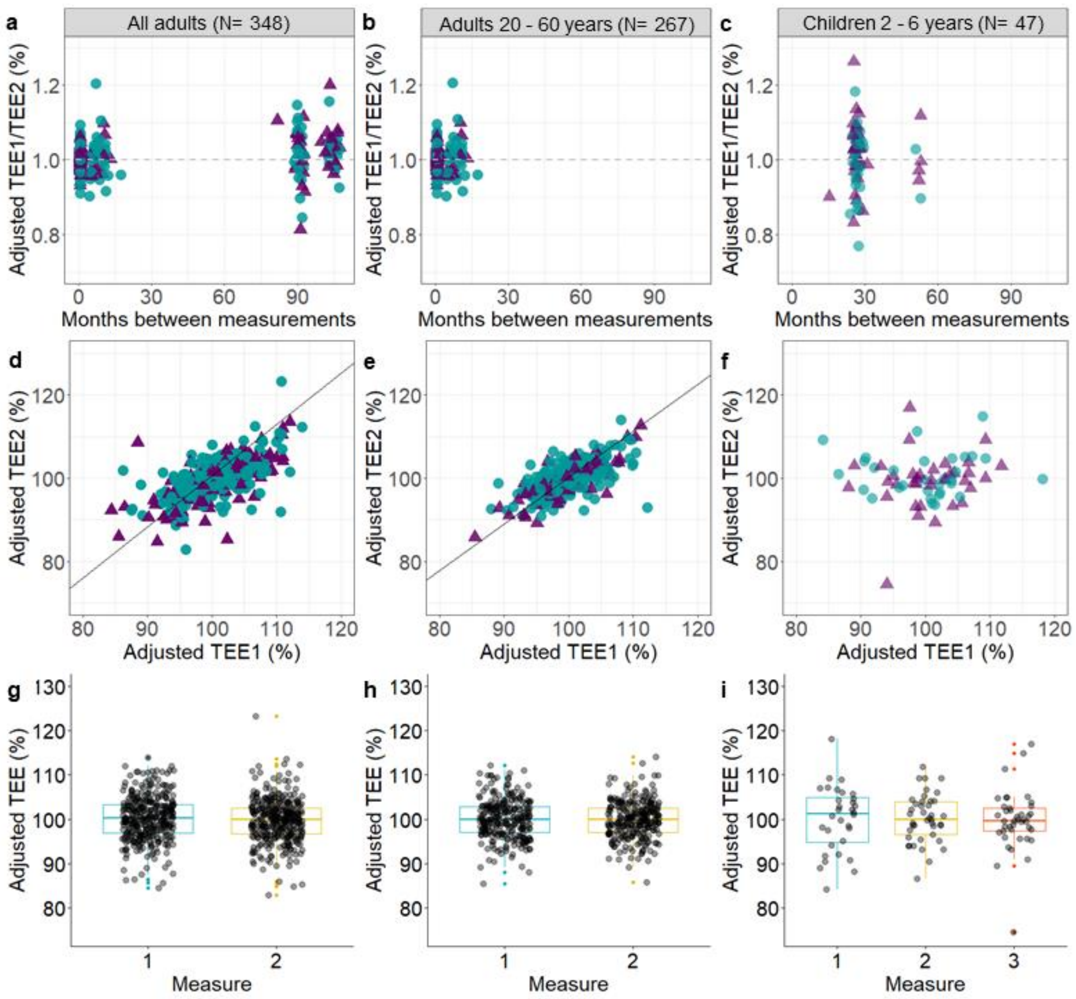

Supplementary Fig. 1. Repeatabilitiy of total energy expenditure (TEE) measurements. a-c Relationship between adjusted TEE1/TEE2 and the time between measuremnts. Dashed lines indicate where adjusted TEE1 and TEE2 are identical (at 1.0). **d-f** Relationship between adjusted TEE1 and TEE2 (blue circles are females and purple triangles are males, lines show reduced major axis regression line). **d** Adjusted TEE1 correlated with adjusted TEE2 (Pearson's product-moment correlation: $r_p = 0.63$, $P < 0.0001$) in all adults. **e** Adjusted TEE1 correlated with adjusted TEE2 (Pearson's product-moment correlation: $r_p = 0.69$, $P < 0.0001$) in adults aged 20 – 60 y. **g-i** Comparison of adjusted TEE between repeated TEE measurements (grey circles show individual data points). Boxplot center line represents the median, lower and upper box limits represent the 1st and 3rd quartile (IQR), whiskers minimum and maximum values of nonoutlier data (±1.5 × IQR) and outliers are indicated by small filled circles. **g** Adjusted TEE1 differed statistically from adjusted TEE2 (Paired t test: t = 2.472, df = 347, P = 0.013, Cohen's d = 0.13; TEE of 348 adults was measured twice, resulting in a total of 696 TEE measurements). **h** Adjusted TEE1 did not differ from adjusted TEE2; TEE of 267 adults was measured twice, resulting in a total of 534 TEE measurements. **i** Adjusted TEE measurements did not differ between measurements. Measure 1 is equivalent to age 2 (N = 32 TEE measurements), measure 2 to age 4 (N = 41 TEE measurements), and measure 3 to age 6 (N = 41 TEE measurements).

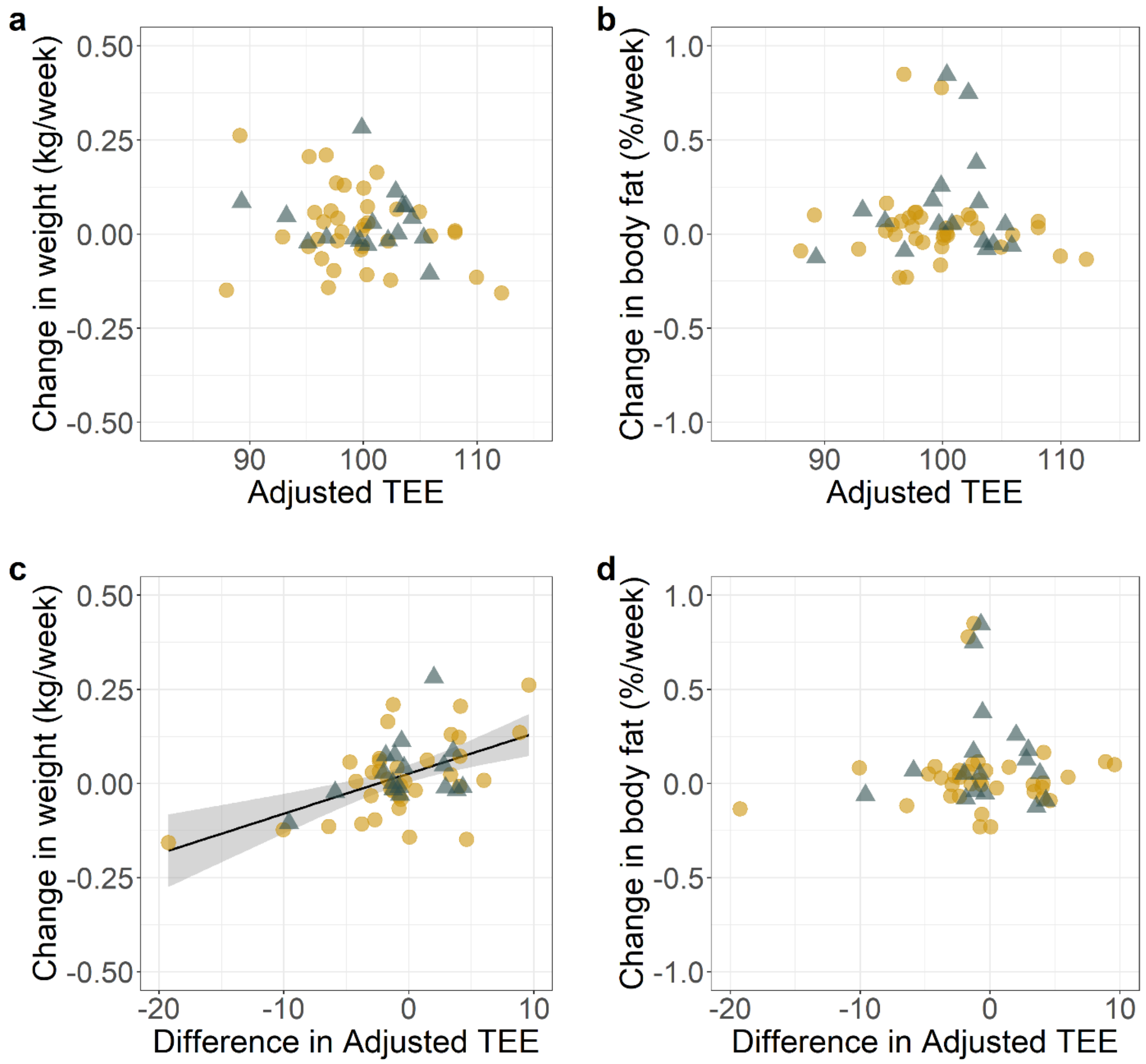

Supplementary Fig. 2. Relationship between adjusted total energy expenditure (TEE), the difference in adjusted TEE between measurements and changes in body weight and body fat percentage. Relationship between adjusted TEE (MJ/d; adjusted for FFM, FM, age and sex) at the first measurement and **a** changes in body weight and **b** changes in body fat percentage until the second TEE measurement. Relationship between the difference in adjusted TEE between measurements and **c** changes in body weight (linear regression line is shown and shaded area indicates 95% CI) and **d** changes in body fat percentage until the second TEE measurement in a subset including adults 20 – 60 y (N = 53 subjects) for which TEE measurements that were at least 4 wk apart (time interval: 29.1±12.8 weeks; yellow circles present females and grey triangles present males).

Supplementary Note 2

Does TEE predict subsequent changes in weight or body composition?

To examine if TEE predicts changes in body weight or body fat percentage, we calculated changes in body weight (kg/wk), FFM (kg/wk), FM (kg/wk), body fat percentage (% FM/wk) and TEE (MJ/wk) as the change in each measurement divided by the time that elapsed between both TEE measurements. Among adults 20 – 60 y, mean change in body weight between the two TEE measurements was -0.04 ± 0.21 kg/wk (range: -0.98 – 0.84 kg/wk), and the mean change in % body fat was 1.05 ± 1.26 % fat/wk (range: -6.57 – 6.16 % fat/wk). There was a strong negative correlation between change in FFM and change in FM (r_p = -0.97, P < 0.0001; Supplementary Fig. 3a). Change in adjusted TEE was negatively correlated with change in FM (r_p = -0.53, P < 0.0001; Supplementary Fig. 3b) and positively with change in FFM (r_p = 0.56, P < 0.0001; Supplementary Fig. 3c). Change in body weight primarily reflected loss of FFM (r_p = 0.34, P < 0.0001), as very few subjects exhibited an increase in FFM between time 1 and time 2 (Supplementary Fig. 3a). Age was not correlated with change in FFM (r_p = -0.003, P = 0.95; Supplementary Fig. 3d).

Changes in weight, FM and body fat percentage per week were smaller when using a subset of adults 20 – 60 y (N = 53 individuals) for which the interval between repeated TEE measurements was longer than 4 weeks (range: 4.43 – 68.57 weeks) (change in weight: 0.02 ± 0.09 kg/week, range: -0.15 – 0.28 weeks; change in FM: 0.07 ± 0.19 kg/week, range: -0.16 – 0.70 kg/week; change in % fat: 0.07 ± 0.23 % fat/week, range: -0.23 – 0.85 % fat/week) compared to when TEE measurements were collected within 4 weeks (range: 1.85 – 3.71 weeks) of each other (change in weight: -0.05 ± 0.22 kg/week, range: -0.97 – 0.84 weeks; change in FM: 0.91 ± 0.92 kg/week, range: -4.73 – 4.70 kg/week; change in % fat: 1.29 ± 1.29 % fat/week, range: -6.57 – 6.15; N = 207 individuals). Change in body weight did not correlate with change in FFM (r_p = 0.06, P = 0.65), and very few subjects exhibited an increase in FFM between time 1 and time 2 (Supplementary Fig. 3e). The mean change in % body fat was 0.07 ± 0.23 % fat/wk (range: -0.23 – 0.85 % fat/wk). There was a negative correlation between change in FFM and change in FM (r_p = -0.86, P < 0.0001; Supplementary Fig. 3e). Change in adjusted TEE was negatively correlated with change in FM (r_p = -0.48, P = 0.0002; Supplementary Fig. 3f) and positively correlated with change in FFM (r_p = 0.74, P < 0.0001; Supplementary Fig. 3g). Age was not correlated with change in FFM (r_p = -0.22, P = 0.10; Supplementary Fig. 3h).

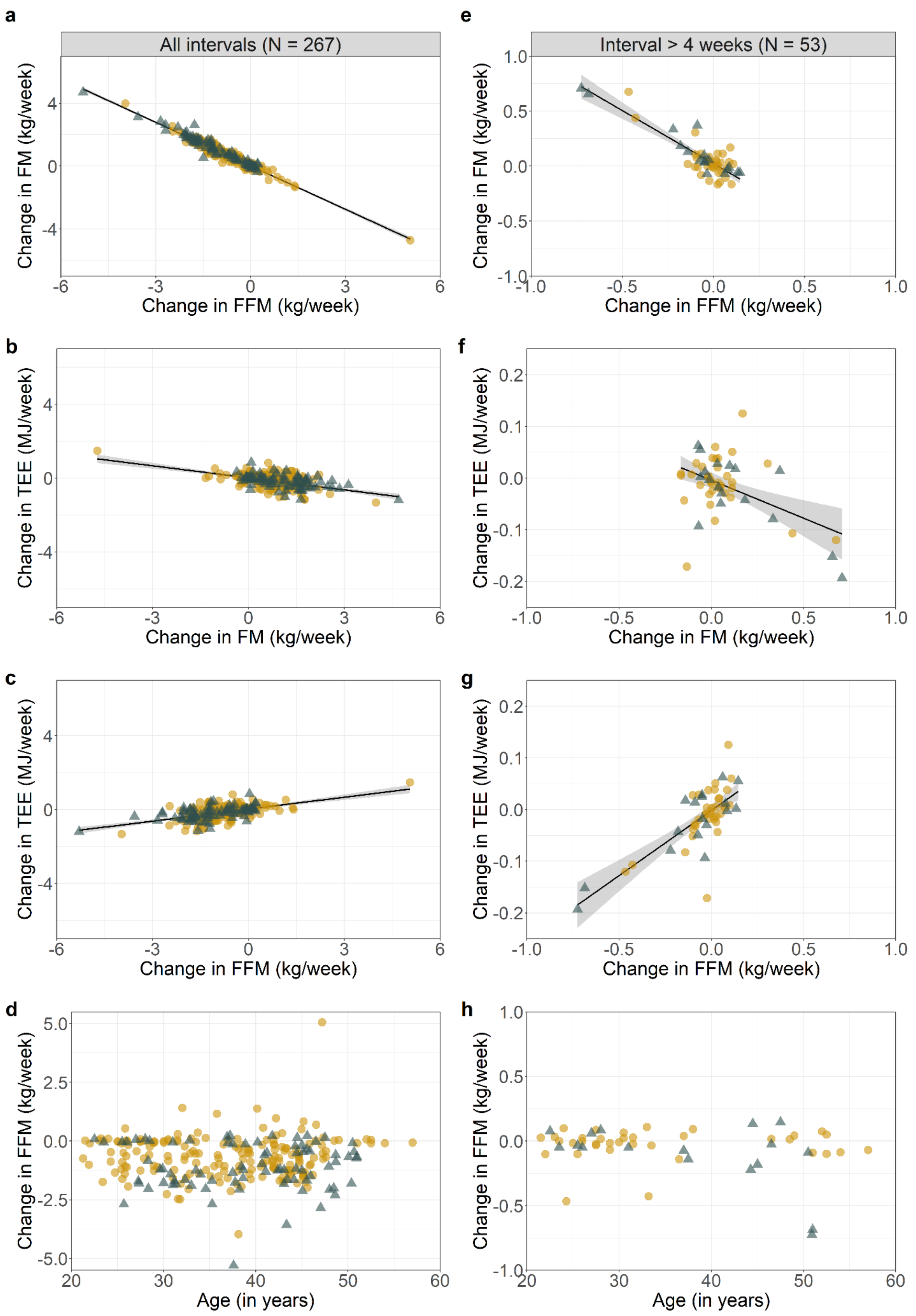

a
All intervals (N = 267)
Change in FM (kg/week)
Change in FFM (kg/week)
e
Interval > 4 weeks (N = 53)
Change in FM (kg/week)
Change in FFM (kg/week)
b
Change in TEE (MJ/week)
Change in FM (kg/week)
f
Change in TEE (MJ/week)
Change in FM (kg/week)
c
Change in TEE (MJ/week)
Change in FFM (kg/week)
g
Change in TEE (MJ/week)
Change in FFM (kg/week)
d
Change in FFM (kg/week)
Age (in years)
h
Change in FFM (kg/week)
Age (in years)

Supplementary Fig. 3. Relationship between changes in body composition and changes in total energy expenditure (TEE) and age-related changes in FFM. a - d Changes in body composition between repeated measurements in all adults aged 20 – 60 years (N = 267 subjects; time interval: 7.4 ± 12.2 weeks). **e - h** Changes in body composition between repeated measurements in a subset of adults (N = 53 subjects) for which the interval between repeated TEE measurements was longer than 4 weeks (time interval: 29.1 ± 12.8 weeks). **a, e** Relationship between changes in fat-free mass (FFM) and fat-mass (FM). **b, f** Relationship between changes in FM and adjusted TEE (adjusted for FFM, FM, age and sex). **c, g** Relationship between changes in FFM and adjusted TEE. **d, h** Relationship between age at the midpoint between both measurements and changes in FFM (yellow circles present females and grey triangles present males). **a - c** and **e - g** lines represent linear regression lines and the shaded areas indicate the 95% confidence intervals.

Supplementary Table 1. Results of multiple regression models to assess which factors influence total energy expenditure (TEE). We included fat-free mass (FFM), fat mass (FM), age and sex (female vs male) as explanatory variables. We ln-transformed TEE, FFM, and FM for these models. **a** Results for the model including all adults (N = 348 individuals), **b** results for the model including only adults aged 20 - 60 years (N = 267 individuals), and **c** results for the model including children aged 2 - 6 years (N = 47 individuals).

		Estimate	SE	t	P	Adjusted R^2
**a** All adults	Intercept	-0.776	0.137	-5.667	<0.0001	0.654
	lnFFM	0.820	0.038	21.531	<0.0001	
	lnFM	0.002	0.014	0.128	0.898	
	Age	-0.003	0.000	-10.057	<0.0001	
	Sex[Male]	-0.010	0.015	-0.634	0.526	
**b** Adults 20-60 years	Intercept	-0.657	0.147	-4.483	<0.0001	0.668
	lnFFM	0.771	0.040	19.505	<0.0001	
	lnFM	-0.014	0.014	-0.955	0.340	
	Age	0.001	0.001	1.039	0.299	
	Sex[Male]	0.003	0.016	0.167	0.867	
**c** Children 2-6 years	Intercept	-0.499	0.175	-2.851	0.0052	0.765
	lnFFM	0.820	0.086	9.579	<0.0001	
	lnFM	0.010	0.022	0.465	0.643	
	Age	-0.008	0.013	-0.643	0.521	
	Sex[Male]	0.042	0.021	2.043	0.0435	

The IAEA DLW Database Consortium authorship

This group authorship contains the names of people whose data were contributed into the IAEA DLW database by the analysis laboratory but they later could not be traced, or they did not respond to emails to assent inclusion among the authorship. The list also includes some researchers who did not assent inclusion to the main authorship because they felt their contribution was not sufficient to merit authorship.

Dr. John Speakman
Center for Energy Metabolism and Reproduction, Shenzhen Institutes of Advanced Technology, Chinese Academy of Sciences, Shenzhen, China
Institute of Biological and Environmental Sciences, University of Aberdeen, Aberdeen, UK

Dr Stefan Branth
University of Uppsala, Uppsala, Sweden

Dr Niels C. De Bruin
Erasmus University, Rotterdam, The Netherlands

Dr Lisa H. Colbert
Kinesiology, University of Wisconsin, Madison, WI,

Dr. Richard Cooper
Department of Public Health Sciences, Parkinson School of Health Sciences and Public Health, Maywood, IL, USA

Dr Alice E. Dutman
TNO Quality of Life, Zeist, The Netherlands

Sölve Elmståhl
Lund University, Lund, Sweden

Dr Mikael Fogelholm
Dept of Food and Nutrition, Helsinki, Finland

Dr Tamara Harris
Aging, NIH, Bethesda, MD,

Dr Rik Heijligenberg
Academic Medical Center of Amsterdam University, Amsterdam, The Netherlands

Dr Hans U. Jorgensen
Bispebjerg Hospital, Copenhagen, Denmark

Dr Christel L. Larsson

University of Gothenburg, Gothenburg, Sweden

Dr Margaret McCloskey
Royal Belfast Hospital for Sick Children, Belfast, Northern Ireland

Dr Gerwin A. Meijer
Maastricht University, Maastricht, The Netherlands

Dr Daphne L. Pannemans
Maastricht University, Maastricht, The Netherlands

Dr Renaat M. Philippaerts
Katholic University Leuven, Leuven, Belgium

Dr Elisabet M. Rothenberg
Göteborg University, Göteborg, Sweden

Dr Sabine Schulz
University of Maastricht, Maastricht, The Netherlands

Dr Amy Subar
Epidemiology and Genomics, Division of Cancer Control, NIH, Bethesda, MD,

Dr Minna Tanskanen
University of Jyväskilä, Jyväskilä, Finland

Dr Ricardo Uauy
Institute of Nutrition and Food Technology (INTA), University of Chile, Santiago Chile.

Dr Rita Van den Berg-Emons
Maastricht University, Maastricht, The Netherlands

Dr Wim G. Van Gemert
Maastricht University, Maastricht, The Netherlands

Dr Erica J. Velthuis-te Wierik
TNO Nutrition and Food Research Institute, Zeist, The Netherlands

Dr Wilhelmine W. Verboeket-van de Venne
Maastricht University, Maastricht, The Netherlands

Dr Jeanine A. Verbunt
Maastricht University, Maastricht, The Netherlands
