# Supplementary material for: Total energy expenditure is repeatable in adults but not associated with short-term changes in body composition
Source: Nat Commun. 2022 Jan 10;13:99. doi: 10.1038/s41467-021-27246-z (PMC8748652; doi:10.1038/s41467-021-27246-z)
Supplement: Supplementary file 3 — Reporting Summary [file 41467_2021_27246_MOESM3_ESM.pdf]

nature portfolio

Corresponding author(s): Rebecca Rimbach

Last updated by author(s): Oct 11, 2021

Reporting Summary

Nature Portfolio wishes to improve the reproducibility of the work that we publish. This form provides structure for consistency and transparency in reporting. For further information on Nature Portfolio policies, see our Editorial Policies and the Editorial Policy Checklist.

Statistics

For all statistical analyses, confirm that the following items are present in the figure legend, table legend, main text, or Methods section.

n/a	Confirmed	
☐	☒	The exact sample size (*n*) for each experimental group/condition, given as a discrete number and unit of measurement
☐	☒	A statement on whether measurements were taken from distinct samples or whether the same sample was measured repeatedly
☐	☒	The statistical test(s) used AND whether they are one- or two-sided<br>*Only common tests should be described solely by name; describe more complex techniques in the Methods section.*
☐	☒	A description of all covariates tested
☐	☒	A description of any assumptions or corrections, such as tests of normality and adjustment for multiple comparisons
☐	☒	A full description of the statistical parameters including central tendency (e.g. means) or other basic estimates (e.g. regression coefficient) AND variation (e.g. standard deviation) or associated estimates of uncertainty (e.g. confidence intervals)
☐	☒	For null hypothesis testing, the test statistic (e.g. *F*, *t*, *r*) with confidence intervals, effect sizes, degrees of freedom and *P* value noted<br>*Give P values as exact values whenever suitable.*
☐	☒	For Bayesian analysis, information on the choice of priors and Markov chain Monte Carlo settings
☒	☐	For hierarchical and complex designs, identification of the appropriate level for tests and full reporting of outcomes
☒	☐	Estimates of effect sizes (e.g. Cohen's *d*, Pearson's *r*), indicating how they were calculated

Our web collection on statistics for biologists contains articles on many of the points above.

Software and code

Policy information about availability of computer code

Data collection	No software was used to collect data.
Data analysis	R (version 3.6.2), R package rptR (version 0.9.22), R package MCMCglmm (version 2.29), R package coda (version 0.19-3). We provide the source code used to perform analysis and output files through the OSF repository (https://osf.io/6q2kz/).

For manuscripts utilizing custom algorithms or software that are central to the research but not yet described in published literature, software must be made available to editors and reviewers. We strongly encourage code deposition in a community repository (e.g. GitHub). See the Nature Portfolio guidelines for submitting code & software for further information.

Data

Policy information about availability of data

All manuscripts must include a data availability statement. This statement should provide the following information, where applicable:

- Accession codes, unique identifiers, or web links for publicly available datasets
- A description of any restrictions on data availability
- For clinical datasets or third party data, please ensure that the statement adheres to our policy

All data supporting the analyses and results in this paper are available from the Doubly Labeled Water Database (https://doubly-labelled-water-database.iaea.org/home, https://www.dlwdatabase.org/) upon reasonable request. Because of subject confidentiality the database is not open access. However, access to components of the data is freely available to perform novel and approved analyses. Details of the application process are available at https://www.dlwdatabase.org. We provide a minimum dataset through the OSF repository to demonstrate the code (https://osf.io/6q2kz/).

Field-specific reporting

Please select the one below that is the best fit for your research. If you are not sure, read the appropriate sections before making your selection.

- [x] Life sciences
- [ ] Behavioural & social sciences
- [ ] Ecological, evolutionary & environmental sciences

For a reference copy of the document with all sections, see nature.com/documents/nr-reporting-summary-flat.pdf

Life sciences study design

All studies must disclose on these points even when the disclosure is negative.

Sample size	We included all individuals in the IAEA DLW database (https://doubly-labelled-water-database.iaea.org/ home) which had their TEE measured at least twice and that were at least 1 year old. This resulted in 696 TEE measurements of 348 adults and 114 TEE measurements of 47 children.
Data exclusions	We excluded TEE measurements from children younger than 1 year because the relationship between TEE and FFM in this age group appears to differ from that for older children.
Replication	One of the main purposes of this study is to assess repeatability of TEE measurements. TEE of adults was measured twice. For 27 children, two TEE measurements were available and for 20 children, three repeated measurements were available.
Randomization	Not applicable, there are not experimental groups.
Blinding	Not applicable, there are not experimental groups.

Reporting for specific materials, systems and methods

We require information from authors about some types of materials, experimental systems and methods used in many studies. Here, indicate whether each material, system or method listed is relevant to your study. If you are not sure if a list item applies to your research, read the appropriate section before selecting a response.

Materials & experimental systems

n/a	Involved in the study	
x		Antibodies
x		Eukaryotic cell lines
x		Palaeontology and archaeology
x		Animals and other organisms
	x	Human research participants
x		Clinical data
x		Dual use research of concern

Methods

n/a	Involved in the study	
x		ChIP-seq
x		Flow cytometry
x		MRI-based neuroimaging

Human research participants

Policy information about studies involving human research participants

Population characteristics	This sutdy is a secondary analysis of a sub-population of DLW data compiled into the IAEA DLW database. The data pertaining to this study involved healthy volunteers of both sexes aged between 2 and 89 years old. The analysis was approved by the database management group and the prior approval posted on the database website (www.dlwdatabase.org).
Recruitment	This sutdy is a secondary analysis of a sub-population of DLW data compiled into the IAEA DLW database and as such we did not recruit participants for this study.
Ethics oversight	All of the studies that provided data into the IAEA DLW database were locally ethically reviewed and approved. The present paper is based on a secondary analysis of these compiled data and such analyses do not require ethical permission.

Note that full information on the approval of the study protocol must also be provided in the manuscript.
